# Pregnancy diet and associated outcomes in the Avon Longitudinal Study of Parents and Children

**DOI:** 10.1093/nutrit/nuv053

**Published:** 2015-09-22

**Authors:** Pauline M. Emmett, Louise R. Jones, Jean Golding

**Affiliations:** *P.M. Emmett* and *J. Golding* are with the Centre for Child and Adolescent Health, School of Social and Community Medicine, University of Bristol, Bristol, UK. L.R. Jones is with the School of Social and Community Medicine, University of Bristol, Bristol, UK.

**Keywords:** ALSPAC, childhood diet, diet during pregnancy, FFQ, fish, folate, iodine, iron, magnesium, mercury, n-3 polyunsaturated fatty acids, neurocognitive development, potassium

## Abstract

All publications covering diet during pregnancy that stemmed from the Avon Longitudinal Study of Parents and Children were reviewed. Diet was assessed using a food frequency questionnaire. Socioeconomic background, maternal mental health, and the health and development of the offspring were assessed using a variety of methods, such as direct measurement, self-completion questionnaires, and assays of biological samples. Differences in diet, including specific food and nutrient intakes and dietary patterns, were associated with maternal educational attainment, smoking habits, and financial difficulty. There were marginal intakes, compared with recommendations, of the key nutrients iron, magnesium, potassium, and folate. Maternal diet during pregnancy was predictive of offspring diet during childhood. There were independent associations between prenatal fish consumption and lower frequency of maternal depressive and anxiety symptoms, as well as lower frequency of intrauterine growth retardation. Consistent evidence that fish consumption during pregnancy benefited the neurocognitive development of the child was also found. Two constituents of fish, n-3 polyunsaturated fatty acids and iodine, were associated with these benefits in children. The findings from the Avon Longitudinal Study of Parents and Children strengthen the recommendation to eat fish regularly during pregnancy.

## INTRODUCTION

Maternal nutrition periconception and during pregnancy is important for the health, growth, and development of the fetus and the newborn infant. There is considerable interest in nutrition during pregnancy because of the fetal origins theory of adult disease. This theory hypothesizes that term infants who are small for gestational age have an increased risk of cardiovascular disease and type II diabetes in adulthood and that this is due to undernutrition of the fetus. The original work was based on the follow-up of historic cohorts for which records of birth weight and early growth were available, but there was no information about the nutritional status of the mothers during pregnancy in these studies.^1^ There has been extensive research following from the original hypothesis in the area of “early nutrition programming.”^2^ This “programming” suggests that an under- or oversupply of a particular nutrient or nutrients at a critical or sensitive period of development may have long-term effects on the structure or function of specific organs or systems in the offspring. The diet may also contain toxic substances that may affect development (e.g., mercury). The sensitive period could be at various stages in utero or in early or later postnatal development and is likely to vary according to the structure being developed.

The fetus may be most susceptible to poor nutrition during the first trimester, as this is the period of rapid cell differentiation and development of embryonic systems and organs.^3^ In fact, the period of greatest risk for most birth defects is in the first few weeks after conception when a mother may not be aware that she is pregnant. For example, it has been found that excessive intakes of vitamin A or low intakes of folate can be teratogenic at this critical period during the early weeks of pregnancy when the first embryonic processes occur, resulting in an assortment of birth defects. This has led to dietary recommendations for pregnancy that limit concentrated sources of vitamin A and stress the importance of periconceptional folic acid supplementation, which has been shown to be protective against neural tube defects.^4^

There is evidence that optimal fetal neurodevelopment is dependent on specific nutrients supplied by the mother mainly from dietary sources; these may include long-chain polyunsaturated fatty acids (LC-PUFAs) such as docosahexaenoic acid (DHA), an n-3 fatty acid, and arachidonic acid, an n-6 fatty acid. These fatty acids are building blocks for fetal retina and brain cells. The fatty acid composition of the cell membrane influences the stability, fluidity, and function of many cell types through effects on gene expression and tissue differentiation.^5^ The major source of n-3 PUFAs in human diets is seafood. It is also possible that fish intake during pregnancy influences neurodevelopment via a mechanism not related to its fatty acid content; other active substances include its vitamin D and iodine content or its possible contamination with mercury. The evidence is in favor of an important in utero programming effect on brain development.^2^

The in utero supply of nutrients or toxins may particularly affect epigenetic factors such as DNA methylation, which are associated with gene expression and thus with the intimate development of the organism.^6^ This may be the mechanism responsible for prenatal programming.

In the developed world, malnutrition is not usually a problem, but there is the question as to the best diet to recommend to expectant mothers during pregnancy in order to maximize the health and development of themselves and their offspring. These recommendations should be evidence-based, and it is becoming widely recognized that birth cohort studies that start in or before pregnancy and then follow the offspring and the wider family can make an important contribution to the evidence.^7^ The Avon Longitudinal Study of Parents and Children (ALSPAC) is one of the few such studies in the world that has followed a population cohort from the pregnancy of the mother through to the adulthood of the offspring. It is unique in collecting dietary information from mothers, their partners, and their offspring. This paper reviews the publications that have used ALSPAC data to report on diet during pregnancy relative to the growth and development of the offspring, as well as to some maternal outcomes.

## LITERATURE AND STUDY METHODS

### Literature

All articles using ALSPAC data that were published up until the end of 2013 and reported on relationships with mothers’ diets during pregnancy were reviewed. This narrative review includes 38 articles (listed in Table 1)^8–45^: 11 with maternal outcomes, 6 with fetal or birth outcomes, and 26 with childhood outcomes.

**Table 1:** Characteristics of the ALSPAC articles included in the present review in the order of presentation in the results

Reference	Dietary input/input	Focus of paper	Age/timing of outcome
Rogers et al. (1998)^8^	Whole diet	Nutrients, food groups	Pregnancy
Northstone et al. (2008)^9^	Dietary patterns	SEB	Pregnancy
Golding et al. (2013)^10^	Whole diet	Blood mercury levels	Pregnancy
Taylor et al. (2013)^11^	Whole diet	Blood lead levels	Pregnancy
Golding et al. (2009)^12^	Fish/seafood intake	Depressive symptoms	Mother during pregnancy and postnatally
Vaz et al. (2013)^13^	Fish/seafood intake	Anxiety symptoms	Mother during pregnancy and postnatally
	Dietary patterns		
Micali et al. (2012)^14^	Pregnancy diet	Eating disorders	Pregnancy
Rogers et al. (1998)^15^	Whole diet	Smoking, financial difficulty, birth weight	Pregnancy, infant at birth

Lawlor et al. (2010)^16^	Gestational diabetes	Macrosomia	Infant at birth
		Obesity	Child 9–11 y
Rogers et al. (2004)^17^	Fish/seafood intake	Birth weight, IUGR, length of gestation	Infant at birth
North et al. (2000)^18^	Vegetarian diet	Hypospadias	In utero
Leary et al. (2005a)^19^	Whole diet	Height, leg length	Child 7 y
Leary et al. (2005b)^20^	Whole diet	Blood pressure	Child 7 y
Leary et al. (2013)^21^	Whole diet	Blood pressure	Child 15 y
Brion et al. (2008)^22^	Dietary and supplementary iron	Blood pressure	Child 7 y
Tobias et al. (2005)^23^	Whole diet	Bone density	Child 9 y
Lawlor et al. (2013)^24^	Vitamin D status	Bone density	Child 9 y
Macdonald-Wallis et al. (2010)^25^	Maternal pre-pregnancy	Bone mass	Child 9 y
	BMI		
Williams et al. (2001)^26^	Oily fish	Visual development	Child 3.5 y
Daniels et al. (2004)^27^	Fish/seafood	Communication skills	Child 18 mo
Hibbeln et al. (2007)^28^	Fish/seafood	Development of skills, behavior, IQ	Child 42 mo, 7 y, 8 y
Koletzko et al. (2011)^29^	FADS genes	Blood fatty acids	Pregnancy
Lattka et al. (2012)^30^	FADS genes	Cord blood fatty acids	Infant at birth
Steer et al. (2012)^31^	FADS genes	Blood fatty acids	Pregnancy, birth, child 7 y
Steer et al. (2014)^32^	Fatty acids in maternal	IQ	Child 8 y
	blood		
Waylen et al. (2009)^33^	Fish	Externalizing behavior	Child 7 y
Bath et al. (2013)^34^	Iodine during pregnancy urine	IQ and reading	Child 8 y, 9 y
Bonilla et al. (2012)^35^	Vitamin B_12_ (diet)	IQ	Child 8 y
Newson et al. (2004)^36^	Fatty acid levels in maternal and cord blood	Asthma, eczema	Child 18 mo, 3 y
Shaheen et al. (2004)^37^	Cord trace minerals	Asthma, eczema	Child 18 mo, 3 y
Shaheen et al. (2009)^38^	Dietary patterns	Asthma, eczema	Child 18 mo, 3 y, 7 y
Granell et al. (2008)^39^	Pregnancy folate, *MTHFR* genotype	Allergy, atopy, asthma	Mother and child 7 y
Davey Smith et al. (2007)^40^	Maternal prepregnancy BMI, paternal BMI	Obesity	Child 7 y
Fraser et al. (2010)^41^	Maternal prepregnancy weight, weight gain	Obesity, CVD outcomes	Child 9 y
Lewis et al. (2009)^42^	Pregnancy folate, *MTHFR* genotype	Body composition, obesity	Child 9 y
de Lauzon-Guillain et al. (2013)^43^	Fruit and vegetable	Child’s fruit and vegetable intake	Child 2 y, 3 y, 4 y
Jones et al. (2014)^44^	Dietary variety	Child’s dietary variety	Child 2 y, 3 y, 4 y
Brion et al. (2010)^45^	Whole diet	Childhood diet	Child 10 y

*Abbreviations*: BMI, body mass index; CVD, cardiovascular disease; FADS, fatty acid desaturase; IQ, intelligence quotient; IUGR, intrauterine growth retardation; MTHFR, methyltetrahydrofolate reductase; SEB, socioeconomic background.

### Subjects

The ALSPAC was set up to investigate the ways in which genes and the environment interact to affect health, behavior, and development as children grow from the time of gestation through infancy and childhood to adulthood.^46^^,^^47^ It was designed as an observational birth cohort study, recruiting pregnant women residing in 3 Bristol-based health districts of the county of Avon, in the southwest of England, with an expected delivery date between April 1991 and December 1992 (n = 14 541 pregnancies, of which 14 015 survived to the third trimester). Most women were recruited before 12 weeks of pregnancy but some presented later in pregnancy. The recruitment area covered the city of Bristol and surrounding urban and rural areas, including towns and villages with some industrial areas and farming communities (total population in 1991, ∼0.9 million). Ethical approval for the study was obtained from the ALSPAC Law and Ethics Committee and the local research ethics committees.

From the outset, one of the focuses for data collection during pregnancy concerned the different features of the mother’s diet. With advice from David Horrobin,^48^ it was decided that it would be important to measure the different types of fat consumed by the mother and to ensure that the women consuming different amounts of n-3 LC-PUFA could be identified in the study. Because fish, particularly fatty fish, are a major source of these fatty acids and consumption is likely to be periodic (i.e., less than daily), it was decided that a food frequency questionnaire (FFQ) would be the most appropriate methodology to use. Furthermore, it was recognized that it would be important also to measure the diet in as much detail as possible so that intakes of various nutrients and other foods could be assessed.

The indicators of socioeconomic background (SEB) of the family at recruitment—such as highest maternal educational attainment, age, and housing tenure—ethnicity, smoking status, and prepregnancy body mass index (BMI) of the mothers are shown in Table 2. The cohort was population based and broadly representative of the population of women with children aged <1 year in Avon in 1991, as covered in the national census.^46^ There was a relatively low proportion of ethnic minority women recruited (2.2% in ALSPAC, whereas in the United Kingdom as a whole the prevalence of nonwhite women with children aged <1 y was 7.6% and in Avon in particular it was 4.1%). The children in ALSPAC (n = 14 062 at birth; n = 13 988 alive at 1 y) have been followed using parental and teacher self-completion questionnaires, medical records, health service data, and educational records and by hands-on assessment at dedicated research clinics. Table 2 shows the SEB of the mothers who completed the dietary assessment during pregnancy and those of children who completed the intelligence quotient (IQ) assessment at age 8 years in comparison with the originally recruited mothers. The retained mothers have higher educational attainment, are older, and have more favorable health indicators than the mothers who did not continue in the study or those whose children did not take part in the IQ assessment. A proportion of children born in the last 6 months of the recruitment phase (equivalent to 10% of the whole cohort) were selected to take part in a substudy known as Children in Focus. These parents were invited to bring their child to research clinics 10 times between the ages of 4 months and 5 years (n = 1432 ever attended).

**Table 2:** Socioeconomic characteristics of the women recruited to the ALSPAC (excluding those with miscarriages)

Characteristic	Whole cohort (n = 14 015)	Women who completed FFQ during pregnancy (n = 12 418)		Mothers of children who completed IQ assessment at 8 y of age (n = 7354)	
	N (%)	N (%)	*P*-value^a^	N (%)	*P*-value^b^
Highest educational attainment			<0.001		<0.001
No academic qualifications at age 16 y	3703 (26.4)	3703 (29.9)		1473 (20.1)	
Academic qualification at age 16 y^c^	4269 (30.4)	4267 (34.4)		2370 (32.2)	
Qualifications beyond age 16 y^c^	4354 (31.1)	4354 (35.1)		2953 (40.2)	
Missing	1691 (12.1)	78 (0.6)		558 (7.6)	
Maternal age at birth (years)			<0.001		<0.001
<20	655 (4.7)	485 (3.9)		124 (1.7)	
20–24	2682 (19.1)	2226 (17.9)		909 (12.4)	
25–29	5369 (38.3)	4842 (39.0)		2799 (38.1)	
30–34	3808 (27.2)	3559 (28.7)		2340 (31.8)	
≥35	1382 (9.9)	1289 (10.4)		872 (11.9)	
Missing	119 (0.8)	17 (0.1)		310 (4.1)	
Housing tenure			<0.001		<0.001
Mortgaged or owned	9547 (68.1)	9027 (72.8)		5707 (77.6)	
Council and housing association rented	2089 (14.9)	1737 (14.0)		612 (8.4)	
Privately rented or other	1399 (10.0)	1205 (9.7)		518 (7.0)	
Missing	980 (7.0)	433 (3.5)		517 (7.0)	
Ethnicity			<0.001		<0.001
White	11 914 (85.0)	11 914 (96.1)		6648 (90.4)	
Nonwhite	319 (2.3)	319 (2.6)		122 (1.7)	
Missing	1782 (12.7)	169 (1.4)		584 (7.9)	
Smoked in last trimester of pregnancy			<0.001		<0.001
Yes	2407 (17.2)	2407 (19.4)		924 (12.5)	
No	8850 (63.1)	8850 (71.4)		5315 (72.3)	
Missing	2758 (19.7)	1145 (9.2)		1115 (15.2)	
Maternal prepregnancy BMI			<0.001		<0.001
<18.5	571 (4.1)	531 (4.3)		274 (3.7)	
18.5–24.99	8525 (60.8)	8137 (65.6)		4828 (65.7)	
25–29.99	1733 (12.4)	1641 (13.2)		939 (12.8)	
≥30	630 (4.5)	594 (4.8)		332 (4.5)	
Missing	2556 (18.3)	1488 (12.0)		981 (13.3)	

^a^ *t*-test comparing women in the original cohort with those who completed a food frequency questionnaire during pregnancy.

^b^ *t*-test comparing women in the original cohort with the mothers of children who completed intelligence quotient assessment at 8 years of age.

^c^ Academic qualifications are normally taken at set ages in schools in the United Kingdom, but those women who obtained them at different ages were included in these groups.

*Abbreviations:* BMI, body mass index; FFQ, food frequency questionnaire; IQ, intelligence quotient.

### Dietary assessment

#### Food frequency questionnaire during pregnancy

Maternal diet was assessed using an unquantified FFQ completed by the women at 32 weeks gestation.^8^ The questionnaire covered the types of foods/drinks typically consumed in the United Kingdom in the early 1990s and was based on a questionnaire used previously in a neighboring area and on weighed intake data collected from women (nonpregnant) in the local area.^8^ The FFQ was not validated prior to use due to lack of funding; however, the questions about fish consumption were later shown to be associated with both n-3 LC-PUFA^26^ and mercury concentrations in maternal blood.^27^ The FFQ contained questions about the weekly frequency of consumption of 43 different foods and food groups. The women were asked to tick one of the following options for each food as consumed “nowadays”: never or rarely, once in 2 weeks, 1–3 times per week, 4–7 times per week, more than once a day. More detailed questions were asked about the consumption of a further 8 foods usually consumed daily (bread, spreading and cooking fat, milk, coffee, tea, soft drinks, and sugar). There were also questions about the types of some foods (bread, cooking and spreading fats, milk, and soft drinks) and about the ways in which foods were prepared and eaten (whether some or all of the fat was cut off meat, how often food was fried, and how many of the slices of bread eaten in a day were spread with fat). No questions were asked about portion sizes; therefore, standard portions were used for the nutrient estimations (see below). Women were asked to indicate if they considered themselves to be “vegetarian” or “vegan” or not. Data were collected on alcohol and caffeine intake in the women, but the papers concentrating on these topics are not considered in this review.

#### Assessment of nutrients

The FFQ was used to calculate an approximate daily nutrient intake for each mother.^8^ Each food group question was assigned a composition, based on consideration of how commonly various foods included in that food group were likely to be consumed and using an amount equivalent to one portion of that food group suitable for an adult woman. For example, the foods included in calculation of the nutrient content of one portion of leafy green vegetables were 0.4 portions of cabbage, 0.4 portions of Brussels sprouts, and 0.2 portions of spring greens. When a question on food consumption had not been answered, it was assumed that the food was rarely or never eaten. The approximate weekly intake was calculated by multiplying the weekly frequency of consumption of a food/food group by the nutrient content (obtained from the 5^th^ edition of McCance and Widdowson’s *The Composition of Foods* and its supplements^49–58^) of a portion of that food and summing this for all the foods consumed. The weekly frequencies of consumption assumed for each of the options in the questionnaire were: “never or rarely” = 0, “once in 2 weeks” = 0.5, “1–3 times per week” = 2, “4–7 times per week” = 5.5, and “more than once a day” = 10. The question on bread consumption asked how many pieces of bread, rolls, or chapattis were eaten on a normal day. Milk consumption was calculated by summing the likely amount of milk consumed in tea and coffee, with breakfast cereals, on its own, and as flavored milk drinks. The nutrient values obtained were then divided by 7 to convert them to a daily intake. Cut-offs for energy intake were applied based on inspection of a histogram of the intakes, thus eliminating extremely low or high intakes (n = 413 subjects [3.3%] were excluded). Approximate daily intakes were calculated for energy, protein, carbohydrate, fat, saturated fat, monounsaturated fat, polyunsaturated fat, total sugar, free (added) sugars, starch, nonstarch polysaccharide (a measure of fiber), n-3 fatty acids (from fish sources only), 12 vitamins, and 9 minerals. The adequacy of the nutrient intakes was assessed against dietary reference values for pregnant women in the United Kingdom.^59^ The reference nutrient intake (RNI) is the amount at which 97.5% of the population are assumed to have an adequate intake.

#### FFQ used at other times

The pregnancy FFQ was adapted for use in the children and parents at later times throughout the study (partners, usually fathers, were contacted via the mother, not directly). Modifications included the addition of questions about foods often consumed by children (e.g., fish fingers and sweets) and foods becoming more commonly consumed (e.g., vegetarian pies). The frequency categories and method of calculating the nutrients remained the same, but the portion sizes used differed as appropriate to the group being assessed.

#### Assessment of fruit and vegetable intake and diet variety

Fruit and vegetable intake was assessed for the mother and child using FFQs and summing all of the frequencies of fruits and vegetables.^43^ The comparison groups were >3 servings/day vs ≤3 servings/day for fruit and vegetables combined. Consumption of a varied diet was assessed in mother and child by creating a healthy plate variety score^44^ using FFQ data based on the food groups and the number of servings as recommended in the food plate model (former pyramid model) promoted by the US Department of Agriculture.^60^ The purpose of a variety score is to measure variety both within and between food groups.

#### Omega-3 long-chain polyunsaturated fatty acid assessment (from fish and seafood only)

In the FFQ there were 3 questions about fish consumption: “How many times nowadays do you eat: 1) white fish (cod, haddock, plaice, fish fingers, etc.); 2) dark or oily fish (tuna, sardines, pilchards, mackerel, herring, kippers, trout, salmon, etc.); 3) shellfish (prawns, crabs, cockles, mussels etc.).” Portion sizes were based on typical consumption patterns in the United Kingdom, and fatty acid compositions were based on profiles of typical species found in UK waters.^61^ Oily fish consumption was validated by comparison with the erythrocyte fatty acid composition of blood samples obtained during pregnancy.^26^ Intakes of the n-3 LC-PUFAs, eicosapentaenoic acid, and DHA from fish sources only were calculated.

#### Dietary pattern assessment

Principal component analysis was used to identify dietary patterns in the pregnancy diet.^9^ Five components described the underlying dietary patterns of the women. Dietary patterns have the advantage of using the frequencies of foods eaten directly and are not reliant on estimating the portion size and composition of foods and food groups as is the case for nutrient estimations.^9^

#### Food records at age 10 years

When the ALSPAC children were aged 10 years (in 2002–2003) their diet was assessed using 3 1-day food records completed by the child with parental help.^62^ The databank used for nutrient analysis was the same as that used for the FFQ during pregnancy.^49–58^ The average daily nutrient intakes and amounts of various food groups were calculated.^62^

#### Misreporting of dietary intake

Underreporting of energy intake in mothers was determined as reported daily energy intakes of <120% of the mother’s estimated basal metabolic rate.^63^ Maternal basal metabolic rate was estimated by using Schofield’s equations for adults described in the UK Department of Health Report on Social Sciences^59^ and based on age and body mass index (BMI; kg/m^2^). Self-reported prepregnancy weight and height were collected via questionnaire during the pregnancy and used to estimate the woman’s prepregnancy BMI and hence her basal metabolic rate. Child misreporting of energy intake was determined using a method that allows for moderate physical activity and uses standardized equations that account for the age, sex, and weight of the child.^62^^,^^64^

### Socioeconomic and anthropometric factors

Maternal age at delivery was calculated by subtracting the mother’s date of birth from the child’s date of birth. Offspring birth weight was obtained from the medical records, and supine length was measured with a Harpenden neonatometer (Holtain, Dyfed, UK) soon after birth by a trained and validated member of the ALSPAC study team. Length of gestation was assessed from the mother’s last menstrual period, unless the ultrasound estimate differed by 2 weeks or more in which case the ultrasound method was used. The following data were collected using questionnaires during pregnancy: parity, measured as the number of previous pregnancies resulting in a live or stillbirth; ethnic background; maternal smoking status at various time points during pregnancy; housing tenure; mother’s perception of financial difficulties, including difficulty affording food. The mother’s highest educational attainment was used to derive a 5-point scale with the following categories: no academic qualifications; vocational training (hairdressing, catering, etc.); O-level academic examination usually taken at age 16 years or equivalent; A-level academic examination usually taken at age 18 years or equivalent; university degree (it is important to note that, although 16 and 18 years are the usual ages for these qualifications, no restrictions based on the age at which the qualification was obtained were applied). In some analyses and in Table 2 these have been contracted to the following 3 categories: low, less than O-level; medium, O-level qualification or equivalent; high, A-level or higher. The details of all questionnaires used are available on the ALSPAC website.^65^

### Maternal depressive and anxiety symptoms

The Edinburgh Postnatal Depression Scale was designed to exclude symptoms ascribable to somatic effects of pregnancy and childbirth (e.g., weight gain, sleeplessness, and tiredness). It was self-completed by the mother in the questionnaires sent at 18 and 32 weeks gestation and post delivery at 8 weeks and 8 months to assess maternal symptoms of depression.^66^ At similar time points, symptoms of maternal anxiety were assessed using the 8 anxiety items from the Crown-Crisp Experiential Index, a validated self-rating inventory related to free-floating anxiety.^13^ There is no established cut-off for this measure, but women who scored in the top 15% were identified as having a high frequency of anxiety symptoms.^13^

### Analysis of biological samples for fatty acids, vitamins, lead, mercury, and other trace minerals

Biological samples were collected from mothers using 4 methods: 1) routinely at the pregnancy enrollment clinic and, thereafter, whenever the woman had blood taken, a sample was requested for ALSPAC^47^; 2) at their first visit to the antenatal service, women had blood taken in acid-washed vacutainers specifically for trace metal analysis^10^; 3) urine samples were obtained at various stages of pregnancy^34^; 4) cord blood was collected at delivery,^30^ and a piece of the umbilical cord was cut off and frozen.^37^

The percentage of each fatty acid as a proportion of the total fatty acid content of the red blood cell membrane phospholipids was measured by gas chromatography in the laboratory of Scotia Pharmaceuticals.^28^^,^^29^ Serum 25-hydroxyvitamin D concentrations for mothers were measured with high-performance liquid chromatography tandem mass spectrometry with an internal standard and adjusted for season of blood collection.^24^ Whole blood samples were sent to the Centers for Disease Control and Prevention for analysis of maternal whole-blood mercury, lead, selenium, and cadmium.^10^ Urinary iodine concentration (and creatinine to correct for urine volume) was assessed in a subset of stored urine samples from the first-trimester of pregnancy.^34^ A subset of the umbilical cord aliquots was analyzed for a number of trace minerals.^37^

### Child’s stereoacuity

Stereoacuity was assessed at age 3.5 years in the Children in Focus substudy (n = 435 with complete data).^26^ Stereoacuity matures through 3 stages, and the test was designed to show which stage a child had reached. The stereotests were carried out by one orthoptist who was blind to the other data available for each child.

### Child’s anthropometric measurements, bone mass, and blood pressure

Height and weight were measured at the research clinics between the ages of 7 years and 15 years. Fat mass was assessed using bioelectrical impedance with a Tanita leg-to-leg body fat analyzer (Model TBF 305; Tanita, Tokyo, Japan). A Lunar prodigy DXA (dual-energy X-ray absorptiometry) scanner (GE Medical Systems, Madison, WI, USA) was used to measure body composition and provided estimates of total fat mass, lean body mass, and bone mass. Systolic and diastolic blood pressures were measured using a Dinamap 9301 Vital Signs Monitor (Morton Medical, London, UK). Two right arm measurements were recorded using a cuff size appropriate for a child’s upper arm circumference, and an average of the 2 measurements was taken.

### Child’s cognitive development (including intelligence quotient) and behavior

ALSPAC developed a questionnaire-based scale to assess the child’s abilities throughout infancy and early childhood. The scale included items from the Denver Developmental Screening Test^67^ and was used to calculate a continuous score with 4 domains: gross motor skills, fine motor skills, social skills, and communication skills. IQ was measured in a research clinic at age 8 years using a validated age-adjusted shortened version of the Wechsler Intelligence Scale for Children.^68^ Scores were calculated for performance, verbal, and total IQ. The strengths and difficulties questionnaire^69^ was completed by caregivers for their children at various ages. The scale consists of 25 questions with 5 subscales: prosocial, hyperactivity, emotional symptoms, conduct problems, peer problems, and a total difficulties score. When the children were aged 7.9 years, information about behavior and development was also collected from parents and teachers using a questionnaire version of the Development and Well-Being Assessment. This is a validated measure consisting of structured and semistructured questions.^70^ An experienced clinician combined all information about symptoms and their impact using a computerized heuristic to make standardized diagnoses of childhood psychiatric disorders.^71^

### Child’s eczema, asthma, and atopy

At age 2.5 years the children were classified as having eczema if their mother responded positively to the question, “Has your child had an itchy dry skin rash in joints and creases of his/her body (e.g., behind the knees, under the arms) since he/she was 18 months old?”.^37^^,^^38^

Information on wheezing in the child was obtained by asking the mother the following question when the child was aged 6 months and 3.5 years: “In the last 12 months has your child had any periods when there was wheezing with whistling on his/her chest when he/she breathed?”. The information from these 2 periods identified children with 4 patterns of wheezing: nonwheezers, transient infant wheezers, late-onset wheezers, and persistent wheezers.^37^^,^^38^

When the children were aged 7 years, the mothers were asked, “Has your child had any of the following in the past 12 months: wheezing; asthma; eczema; hay fever?”. Children were defined as having current doctor-diagnosed asthma if mothers responded positively to the question, “Has a doctor ever actually said that your study child has asthma?” and positively to one or both of the questions on wheezing and asthma in the past 12 months.^38^^,^^39^

Atopy at age 7 years was defined as a positive reaction to a skin prick test for any of 3 known allergens (cat, grass, and house dust mite). This definition has been shown to detect >95% of children with allergies.^39^

## RESULTS AND COMMENTARY

### Maternal nutrient intakes and dietary patterns

Nutrient intakes calculated for 11 923 pregnant women from the FFQ at 32 weeks gestation^8^ were mostly adequate when compared with recommendations^59^ and were similar to intakes assessed in a representative national sample of nonpregnant women of comparable age who had completed weighed food records over 7 days.^72^ The nutrients most likely to be judged inadequate in the diet of ALSPAC pregnant women were iron (median intake, 10.2 mg; RNI, 14.8 mg), magnesium (median intake, 247 mg; RNI, 270 mg), potassium (median intake, 2553 mg; RNI, 3500 mg), and folate (median intake, 245 µg; RNI, 300 μg). Iron supplements were taken by 22% of the women at 18 weeks of pregnancy and 43% at 32 weeks^8^; fewer women took folate supplements, 9% and 18%, respectively, but this was prior to a national publicity campaign to encourage women to take folate supplements in early pregnancy.

The FFQ data were used to establish dietary patterns underlying the foods consumed by the pregnant women, and these were then related to socioeconomic variables.^9^ There were 5 patterns that together explained 31.3% of the variation of foods in the diet. This is a similar amount of variation to that in a large Swedish validation study assessing dietary patterns obtained from FFQs completed by women vs those obtained from diet records.^73^

The “health-conscious” pattern in ALSPAC loaded positively on brown/wholemeal bread, rice, pasta, fresh fruit, salad, fruit juice, fish, cheese, pulses, and whole grain breakfast cereal and negatively on white bread.^9^ Mothers with higher educational attainment, those who were older, those who did not smoke, and those who were not overweight before pregnancy were more likely to score highly on this pattern.

The “traditional” pattern had high factor loadings on green vegetables, peas, carrots, root vegetables, and potatoes (not French fries). It was not associated with maternal educational attainment but was more likely if there were several children in the household or if the mother was overweight.^9^

The “processed” pattern had high factor loadings for white bread, meat pies, sausages, pizza, eggs, chips and roast potatoes, baked beans, and fried foods. The strongest associations with this pattern were with the mother being aged <20 years, smoking during pregnancy, and having lower educational attainment. Having more children in the household, living in council accommodation (public housing), and reporting financial difficulties were all independently associated with higher scores on this pattern.^9^

The “confectionery” pattern loaded on chocolates, sweets, crisps, biscuits, puddings, and cakes and was associated positively with young maternal age and negatively with mothers being overweight or dieting during pregnancy.^9^

The “vegetarian” pattern had positive loadings on pulses, nuts, meat substitutes, and herbal tea and negative loadings on red meat and poultry. The women with higher scores on this pattern were more likely to be older and to have financial difficulties and less likely to have medium educational attainment or to have other children.^9^

These data suggest that the diet consumed during pregnancy was adequate for most nutrients with the exception of some key micronutrients—namely, iron, magnesium, potassium, and folate. There was strong evidence of social bias in the dietary patterns that describe the diets of ALSPAC women during pregnancy.

### Toxins in the maternal diet

Foods can supply toxins, as well as nutrients, and the developing fetus may be particularly susceptible. For example, high blood concentrations of mercury and lead during pregnancy have been shown to be associated with adverse offspring outcomes. Maternal blood mercury^10^ and lead concentrations^11^ in the ALSPAC were investigated in relation to environmental and dietary exposures. Linear regression was used to determine the contribution to total blood mercury of 103 food/drink types based on *R*^2^ values; maternal diet accounted for 19.8% of the total variation, with 8.75% coming from fish/seafood.^10^ Other components of the diet that contributed positively included wine and herbal teas. Some foods had a negative association with total blood concentrations of mercury; these included white bread, meat pies, and French fries. The study concluded that limiting intake of seafood during pregnancy may have only a small effect on total mercury concentrations but may be detrimental to other outcomes.

Lead concentrations in maternal blood (median, 3.41; range, 0.41–19.14 mg/dL) were slightly higher in the ALSPAC than reported in other developed countries, and the strongest predictor of amounts ≥5 μg/dL was high maternal educational attainment.^11^ Other factors independently associated with increased concentrations of lead were cigarette smoking, alcohol and coffee drinking, and heating the home with a coal fire. There was some evidence that higher dietary iron and calcium intakes were associated with lower concentrations. These data suggest that following the recommendations for a healthy diet and lifestyle during pregnancy may have the added benefit of keeping blood lead concentrations low.

### Dietary associations with maternal psychiatric symptoms

Some aspects of maternal psychiatric symptoms were investigated in relation to the mothers’ diet during pregnancy. The presence of depressive symptoms was assessed using the Edinburgh Postnatal Depression Scale several times during and after pregnancy.^12^ Compared with women consuming seafood frequently (>3 portions per week providing >1.5 g/week n-3 LC-PUFA), those consuming none were more likely to have frequent depressive symptoms at 32 weeks of pregnancy, the same point at which the diet was measured (adjusted odds ratio [OR], 1.54; 95% confidence interval [CI], 1.25–1.89). These associations were weaker for depressive symptoms at other time points, possibly because they were more remote from the dietary measure.

Symptoms of anxiety at 32 weeks gestation were investigated in relation to dietary patterns and intakes of n-3 LC-PUFA from fish.^13^ Women who had high scores on the vegetarian dietary pattern were more likely to have frequent anxiety symptoms (OR, 1.25; 95% CI, 1.08–1.44), whereas those with high scores on the health-conscious and traditional patterns were less likely to have symptoms of anxiety. There was also an independent negative association between fish consumption and anxiety symptoms; women who had no n-3 LC-PUFAs from fish had more anxiety symptoms than those who consumed >1.5 g/week of n-3 LC-PUFAs (OR, 1.53; 95% CI, 1.25–1.87).^13^

Women were assessed by questionnaire for a history of eating disorders (anorexia nervosa or bulimia nervosa) during and before pregnancy; 414 women had reported such a history, and their diets during pregnancy were compared with those of the 9723 women in the cohort without a history of eating disorders or other major psychiatric problems.^14^ Women with a history of eating disorders were 2.8 times (95% CI, 2.1–3.8) more likely to state that they were currently a vegetarian than those without. This was reflected in their lower consumption of meat and higher consumption of soya products and pulses, although there was no evidence of differences in nutrient intakes. The women with a history of eating disorders reported a much higher intake of coffee than women without. The highest of these intakes were above the upper limit of caffeine recommended for pregnant women in the United Kingdom.^73^ Taken together, these studies suggest that maternal psychiatric symptoms are related to small differences in maternal diet, which could be either a cause or an effect of the psychiatric problems.

### Birth outcomes

Some aspects of diet during pregnancy may be associated with adverse birth outcomes. Dietary differences relating to the smoking habits and self-assessed financial difficulties of the women and their associations with birth weight were investigated.^15^ Difficulty affording food was commonly reported by the least educated women and in smokers; 14.9% and 15.6% had difficulty, respectively, compared with only 2.7% of the most educated and 6.5% of nonsmokers. As shown earlier, both smoking status and degree of difficulty affording food were related to dietary patterns. In regard to the quality of the diet in terms of foods and nutrients, smokers had higher intakes of energy, saturated fat, and free sugars but lower intakes of protein and most micronutrients, particularly vitamin C, than nonsmokers. This was a reflection of the types of foods eaten: smokers consumed sausages, pies, chips, and crisps more often and red meat, poultry, fish, green vegetables, salad, and fruit less often. Smokers were also less likely to take supplements of iron or folate. As is common, the infants of smoking mothers had a lower average birth weight than those of nonsmoking mothers.

Women reporting the most difficulty affording food had lower intakes of energy and of most nutrients, particularly vitamin C, zinc, and iron, compared with those with no difficulty. Women with difficulty were more likely to eat meat products than to eat carcass meat, poultry, or fish; they ate French fries more often and green vegetables, salad, fruit, and fruit juice less often than those with no difficulty affording food. There were no differences in supplement use according to degree of financial difficulty.^15^ There was no independent association between birth weight and degree of financial difficulty after accounting for smoking status and adjusting for sex, gestational age, maternal height, parity, and ethnicity. The fact that many of the food habits were similar in smokers and women with financial difficulty but the infants of those with financial difficulty did not show the birth weight deficit of the smokers’ infants suggests that maternal dietary differences are not the main determinant of birth weight differences.

The presence of diabetes in the mother (prepregnancy [n = 40], gestational [n = 53]) was associated with greater mean birth weight and with greater odds for macrosomia (birth weight >4000 g) in the infant^16^; the adjusted ORs for macrosomia were 3.56 (95% CI, 1.53–8.28) for existing diabetes and 5.50 (95% CI, 1.18–10.30) for gestational diabetes. There was a smaller increased risk for macrosomia if the nondiabetic mother had at least 2 episodes of glycosuria detected during the pregnancy (n = 372; adjusted OR, 1.58; 95% CI, 1.18–2.12) compared with mothers with none of these problems (n = 10 123). These data are in agreement with previous studies and suggest that even modest increases in concentrations of glucose in maternal blood during pregnancy are associated with a fetal growth rate that is above the normal range.

An investigation of birth outcomes in relation to fish intake during pregnancy was carried out in the wake of several non-ALSPAC studies looking at associations of maternal fish consumption with birth weight, length of gestation, and size at birth that had shown conflicting results.^17^ It was possible that the fatty acid content of fish was driving any associations found; therefore, the intakes of n-3 LC-PUFAs from fish and the weight of fish eaten were estimated. Preliminary analysis showed that n-3 LC-PUFA intakes were strongly positively associated with maternal educational attainment and negatively with smoking status (both *P* < 0.001). Unadjusted positive associations between fish and n-3 LC-PUFA intakes and both birth weight and length of gestation were not robust to adjustment for child’s sex, maternal smoking, age, parity, and education. However, there was a persistent relationship, after adjustment, between low fish intake and intrauterine growth retardation; there was an OR of 1.37 (95% CI, 1.02–1.84) for being below the 10^th^ percentile of birth weight for sex and gestational age if the mother ate no fish during pregnancy compared with mothers who ate the highest quantity of fish.^17^ The relationship was not changed by removing smokers from the analysis and was stronger for fish than n-3 LC-PUFA intake, so it may be related to some other constituent of fish. These results lend some support to the hypothesis that increasing fish intake during pregnancy may increase the growth rate of the fetus.

The association of maternal diet with the presence of a congenital defect of the penis in boys—namely, hypospadias—was explored.^18^ It had been suggested that high intakes of phytoestrogens may be implicated and that vegetarian diets are likely to contain higher amounts than omnivorous diets. Mothers (of boys) who were vegetarian during pregnancy had a higher risk (adjusted OR, 4.99; 95% CI, 2.10–11.88) of giving birth to a hypospadias-affected boy compared with omnivores who did not take iron supplements in the first half of pregnancy, whereas omnivores who took iron supplements had a marginally higher risk (adjusted OR, 2.07; 95% CI, 1.00–4.32). These results support the hypothesis that phytoestrogens may disrupt the development of the male reproductive system.

### Physical growth, blood pressure, and bone development of the offspring

The investigations made into the relationship between maternal diet during pregnancy and the physical growth, blood pressure, and bone development of the offspring are presented in Table 3. A weak association was found between maternal dietary iron and magnesium intake and height, particularly sitting height; it was greatly attenuated on full adjustment.^19^

**Table 3:** Associations of maternal dietary intakes, iron supplement use, anemia, and vitamin D status during pregnancy with measures of offspring growth, blood pressure, and bone development

Measure in child	Dietary factor	Adjustments applied	Minimally adjusted results	Fully adjusted results	Comments
Height at 7.5 y, n = 6663 singletons^19^	Iron Magnesium Vitamin C	Minimal: child sex, age at measurement, pregnancy energy intake Full: plus maternal age, BMI, height, smoking, SEB, parity, breastfeeding	β = 0.08 (95% CI, 0.05–0.12), *P* < 0.001 β = 0.10 (95% CI, 0.07–0.14), *P* < 0.001 β = 0.05 (95% CI, 0.03–0.08), *P* < 0.001	β = 0.04 (95% CI, 0.01–0.08), *P* = 0.02 β = 0.05 (95% CI, 0.01–0.08), *P* = 0.04 β = 0.02 (95% CI, −0.01 to 0.04), *P* = 0.2	Regression analysis using standard deviation scores. Other maternal dietary factors tested but not associated after full adjustment: energy, carbohydrate, protein, fat, calcium, potassium, retinol, vitamin D, folate, n-3 fatty acids, milk, meat, fish, fruit, and vegetables. Sitting height relationships similar to height. Leg length not associated after full adjustment
Blood pressure (systolic) at 7.5 y, n = 6944 singletons^20^	Carbohydrate quartiles n-3 fatty acid quartiles	Minimal: child sex, age at measurement, pregnancy energy intake Full: plus maternal age, BMI, height, smoking, SEB, birth weight, gestation, child’s current size	β = 0.51 (95% CI, −0.17 to 1.19) β = 0.02 (95% CI, −0.79 to 0.83) β = 0.73 (95% CI, −0.39 to 1.85), *P* = 0.5 β = 0.41 (95% CI, −0.19 to 1.00) β = −0.38 (95% CI, −1.03 to 0.27) β = −0.43 (95% CI, −1.04 to 0.18), *P* = 0.04	β = 0.98 (95% CI, 0.16–1.79) β = 1.00 (95% CI, 0.02–1.97) β = 1.52 (95% CI, 0.17–2.87), *P* = 0.04 β = 1.03 (95% CI, 0.30–1.75) β = 0.20 (95% CI, −0.59 to 0.98) β = 0.47 (95% CI, −0.28 to 1.22), *P* = 0.7	Linear regression analysis using lowest intake quartile as reference. Other maternal dietary factors tested but not associated after full adjustment: protein, fat, calcium, potassium, magnesium, protein/carbohydrate ratio, animal protein. Diastolic blood pressure not associated with any of the dietary variables
Blood pressure (systolic) at 15 y, n = 4723^21^	Carbohydrate quartiles	Adjustments as above plus paternal diet	β = 0.67 (95% CI, −0.31 to 1.65) β = 0.02 (95% CI, −0.76 to 1.59) β = 0.73 (95% CI, −0.90 to 2.34) *P* = 0.5	β = 0.52 (95% CI, −0.51 to 1.57) β = 0.66 (95% CI, −0.58 to 1.90) β = 1.03 (95% CI, −0.69 to 2.75) *P* = 0.3	Statistical methods and dietary factors as above. Paternal diet analyzed in the same way. No associations of systolic or diastolic blood pressure were found with either maternal diet during pregnancy or paternal diet.
Blood pressure (systolic) at 7.5 y^22^ ^n = 7130-7484^	Iron intake (per 50 mg/wk) Iron supplements in any trimester	Minimal: sex, age at measurement Full: plus maternal age, BMI, height, smoking, SEB, birth weight, gestation, child’s BMI	β = −0.41 (95% CI, −1.12 to 0.30) *P* = 0.3 β = −0.72 (95% CI, −1.14 to −0.31) *P* = 0.001	β = 0.17 (95% CI, −0.64 to 0.99) *P* = 0.7 β = −0.63 (95% CI, −1.09 to −0.17) *P* = 0.007	Multiple linear regressions were used. No other dietary factors were assessed. The strongest negative association with offspring blood pressure was in women with anemia who did not take iron supplements
Bone density at 9.9 y,^23^ n = 4588	Magnesium Potassium Folate	Minimal: sex, age at measurement, maternal energy intake, SEB, pubertal stage Full: plus all other dietary variables	Total body bone mineral density BMD (g/cm^2^) β = 4.88 (95% CI, 2.46–7.30) *P* < 0.001 Spinal bone mineral density (g/cm^2^) β = 10.5 (95% CI, 4.9–16.0) *P* < 0.001 Area-adjusted spinal bone mineral content ABMC (g) β = 0.55 (95% CI, 0.16–0.94) *P* = 0.006	Magnesium was the only dietary variable independently related to total body BMD. Potassium was the only dietary variable independently related to spinal BMD. Folate was the only dietary variable independently related to spinal ABMC	Standardized regression coefficients. Other maternal dietary factors tested but not associated after full adjustment: carbohydrate, protein, fat, fiber, calcium, sodium, phosphorus, zinc, iron, retinol, carotene, riboflavin, thiamin, niacin, vitamin C, vitamin D, vitamin E, and n-3 fatty acids. Relationship with magnesium not independent of child’s height. Relationship with potassium not independent of child’s weight. Relationship with folate independent of both height and weight of child. No association of bone measures with folate supplements
Bone density at 9.9 y, n = 3196^2^,^4^	Maternal 25(OH)D concentration in third trimester	Minimal: sex, age at measurement, maternal age Full: plus SEB, smoking, BMI, parity, birth weight, gestation, child size	Difference in spinal bone mineral content (g)/10.0 nmol/L 25(OH)D β = 0.01 (95% CI, −0.15 to 0.17) *P* = 0.9	Difference in spinal bone mineral content (g)/10.0 nmol/L 25(OH)D β = 0.06 (95% CI, –0.03 to 0.16) *P* = 0.2	Linear regression was used. Maternal concentration of 25(OH)D was measured in the third trimester

*Abbreviations*: ABMC, area-adjusted bone mineral content; BMD, bone mineral density; BMI, body mass index; CI, confidence interval; SEB, socioeconomic background; 25(OH)D, 25-hydroxyvitamin D.

For offspring, blood pressure measured at ages 7 years and 15 years revealed no convincing associations with maternal intake of any nutrients during pregnancy (Table 3).^20^^,^^21^ Research in animals had suggested that maternal iron deficiency during pregnancy may be related to offspring blood pressure; therefore, relationships with maternal anemia, iron supplementation, and dietary intake were investigated.^22^ There was no association of dietary iron intake during pregnancy with offspring blood pressure at age 7 years (Table 3). There was some evidence that iron supplementation was associated with slightly lower blood pressure, but after accounting for multivitamin use, no association remained. In women not taking supplements, there was a marginal association between anemia during pregnancy and lower offspring systolic blood pressure (fully adjusted β, −1.48; 95% CI, −3.21 to 0.25 mm Hg; *P* = 0.09).^22^

Bone development was measured at age 9 years. There was evidence that maternal dietary intakes of magnesium, potassium, and folate, but not calcium, were positively associated with measures of bone mass (Table 3).^23^ The proportion of explained variability in child bone mass was very small (i.e., for maternal magnesium intake approximately 1% higher total body bone mass between the upper and the lower tertile). Such very small differences do not suggest that diet during pregnancy plays a major role in bone development. There was also no evidence of an association between maternal vitamin D status during pregnancy and offspring bone mineral content (Table 3).^24^ The suggestion that maternal body fat stores are important for fetal bone mineral deposition was investigated in a further study.^25^ There was no evidence of an independent association between maternal prepregnancy BMI and offspring bone mass at age 9 years once adjustments were made for birth weight and the current height and weight of the child.^25^

These data suggest it is unlikely that pregnancy diet has an important effect on any of the outcomes investigated in this section, although the associations that were found were with nutrients that are most likely to be inadequate in the diet of this population.^8^

### Neurocognitive and behavioral development of the offspring

Brain cell membranes have a high content of n-3 LC-PUFAs, and the predominant sources of these nutrients in the diets of UK residents are fish and seafood. For infants, breast milk is also a good source. In light of this, fish consumption during pregnancy and breastfeeding were investigated in relation to neurocognitive development.^26^ The earliest study of this type in ALSPAC measured stereovision at age 3 years in the Children in Focus substudy (435 full-term children with complete data). Better stereovision in children was related independently both to any breastfeeding and to mothers’ eating oily fish at least once every 2 weeks during pregnancy (OR, 1.57; 95% CI, 1.00–2.45).^26^ In a further analysis, eating oily fish during pregnancy was associated with higher maternal blood concentrations of the n-3 LC-PUFA DHA in a dose–response manner (analysis of variance; *F* = 25.1, df = 2, *P* < 0.001). It is possible, therefore, that DHA is the active factor accounting for the association of prenatal fish consumption with visual development. Visual development was not independently associated with the educational attainment of the mother.

During the time of the ALSPAC, there had been concern about the possible detrimental effects of the mercury content of fish on cognitive development, with pregnant women, especially in the United States, being advised to limit their intake of fish due to the potential for high mercury content in some species.^75^ One study using ALSPAC data^27^ looked at the possible effects of the mercury content of fish on the early development of language and communication skills at ages 15 and 18 months (n = 7421) and found that fish consumption during pregnancy was related to marginally better communication skills in the offspring (fully adjusted vocabulary comprehension score at 15 months: no fish, 68.2 [95% CI, 66.3–70.5]; high fish intake during pregnancy, 71.9 [95% CI, 70.5–73.8]; *P* for trend, 0.03). Mercury concentrations in umbilical cord samples were available for a subset of these infants (n = 1054). The amounts were positively associated with the FFQ assessment of fish consumption during pregnancy, but they were not associated with the developmental outcomes measured.^27^ The authors concluded that further studies would be needed to determine what aspects of fish consumption may explain the beneficial association shown.

A further investigation of developmental outcomes in relation to fish consumption assessed IQ measured at age 8 years.^28^ In total, 12% of women had eaten no fish during pregnancy, 65% ate 1–340 g per week, and 23% ate more than 340 g (at least 3 portions per week). Only 2% of the pregnant women had taken fish oil supplements, so this was unlikely to contaminate the results. Table 4 shows the results of the fully adjusted logistic regression for the risk of the child being in the lowest quartile of the outcome measure. Maternal fish consumption was positively associated with total and verbal but not performance IQ (Table 4). The OR for suboptimal score for verbal IQ was further adjusted for father’s fish intake, and the relationship was only slightly attenuated (OR, 1.39; 95% CI, 1.04–1.86), suggesting that the result is unlikely to be explained by social status. The risk of the child having suboptimal scores for prosocial behavior at age 7 years or fine motor skills or social development at age 42 months were greater when no fish was consumed by the mother during pregnancy (see Table 4).^28^

**Table 4:** Maternal fish intake during pregnancy and the likelihood of offspring having suboptimal cognitive and behavioral outcomes

Outcomes	None vs >340 g/week	1–340 g/week vs	Trend	n
		>340 g/week	*P*-value^a^	
Cognition at 8 years (by WISC)				
Verbal IQ	OR, 1.48 (95% CI, 1.16–1.90)	OR, 1.09 (95% CI, 0.92–1.29)	0.004	5407
Performance IQ	OR, 0.98 (95% CI, 0.76–1.27)	OR, 0.99 (95% CI, 0.84–1.18)	0.902	5042
Full scale IQ	OR, 1.29 (95% CI, 0.99–1.69)	OR, 1.19 (95% CI, 0.99–1.42)	0.039	5000
Behavior at 7 years (parental-completed SDQ)				
Prosocial	OR, 1.44 (95% CI, 1.05–1.97)	OR, 1.16 (95% CI, 0.93–1.44)	0.025	6582
Hyperactivity	OR, 1.13 (95% CI, 0.84–1.53)	OR, 0.91 (95% CI, 0.73–1.12)	0.629	6575
Emotional	OR, 1.09 (95% CI, 0.83–1.44)	OR, 0.96 (95% CI, 0.80–1.17)	0.681	6582
Conduct	OR, 1.21 (95% CI, 0.89–1.64)	OR, 1.01 (95% CI, 0.81–1.25)	0.287	6586
Peer problems	OR, 1.25 (95% CI, 0.96–1.62)	OR, 0.97 (95% CI, 0.80–1.16)	0.175	6581
Child development at 42 months (parental-completed questionnaire)				
Gross motor skills	OR, 0.96 (95% CI, 0.78–1.18)	OR, 0.99 (95% CI, 0.87–1.13)	0.716	7603
Fine motor skills	OR, 1.35 (95% CI, 1.09–1.66)	OR, 1.14 (95% CI, 0.98–1.31)	0.005	7596
Social development	OR, 1.21 (95% CI, 0.98–1.50)	OR, 1.17 (95% CI, 1.01–1.35)	0.038	7592

Reproduced from Hibbeln et al. (2007)^28^ with permission.

^a^Logistic regression adjusted for child’s sex, birth weight, preterm delivery, maternal education and age, housing tenure, smoking during pregnancy, parity, breastfeeding, and 12 nonfish food groups.

*Abbreviations*: CI, confidence interval; IQ, intelligence quotient; OR, odds ratio; SDQ, strengths and difficulties questionnaire; WISC, Wechsler Intelligence Scale for Children.

These 3 studies add weight to the evidence for the beneficial effect of fish consumption during pregnancy on brain development but do not determine the mechanism of the association. In light of these results, various government departments were lobbied to modify their recommendations to pregnant women regarding limiting seafood consumption, with the result that recommended intakes of fish during pregnancy have been increased in various countries, including the United States and Norway.^76^ These publications led to ALSPAC’s involvement in a European Union–funded project, Nutrimenthe,^77^ which investigated the possible mechanisms and genetic underpinning of the relationships between fish consumption and neurocognitive development, in particular exploring the genes involved in the metabolic pathways that elongate and desaturate n-3 and n-6 PUFAs (fatty acid desaturase genes [FADS]). This work considered both maternal and child FADS genotype in relation to LC-PUFAs in maternal and cord blood. First, the program showed, as demonstrated in previous smaller studies, that maternal FADS genotype modulates the concentrations of LC-PUFAs in maternal blood.^29^ The minor alleles in the FADS genes were consistently positively associated with medium-chain n-6 and n-3 PUFAs and negatively associated with n-6 and n-3 LC-PUFAs, including DHA. This suggests that they were less efficient at elongating the fatty acid chain, possibly resulting in a lower supply of DHA to the fetus. The second group of analyses showed that, in relation to cord blood concentrations of PUFAs, both maternal and child genotypes were equally important.^30^ As in the maternal blood, the minor alleles of the maternal FADS genes were associated with higher concentrations of medium-chain PUFAs in cord blood. Associations with n-6 LC-PUFAs were seen for the child’s but not the mother’s FADS genes (negative for the minor alleles) and were much stronger than the associations with n-3 LC-PUFAs. Both maternal and child minor alleles were associated negatively with DHA concentrations in cord blood. It had previously been thought that the fetus was totally reliant on maternal supply of DHA and that fetal metabolism was not associated with DHA synthesis; these results show this not to be the case. However, maternal FADS polymorphisms were not associated with n-6 or n-3 PUFA concentrations in the child’s blood at age 7 years; by this age, there were strong associations of child FADS genes with concentrations of both.^31^

An analysis using maternal LC-PUFA concentrations as a direct predictor of IQ at age 8 years^32^ showed only a very weak relationship with DHA concentrations in maternal blood (full-scale IQ points, −1.52; 95% CI, −2.91 to −0.14; *P* = 0.031 for the lowest quartile of the fatty acid compared with the rest), and some n-6 LC-PUFAs (such as osbond acid [22:5n-6]; full-scale IQ points, −1.95; 95% CI, −3.30 to −0.61; *P* = 0.004 and arachadonic acid [20:4n-6]; full-scale IQ points, −1.54; 95% CI, −2.91 to −0.18; *P* = 0.026) were found to have similar relationships.^32^ From postmortem data, it seems that these n-6 LC-PUFAs may be used in cell membranes in place of DHA if there is a shortage of DHA. The percentage of the variation in IQ explained by these fatty acids was very small (e.g., 0.29% for osbond acid) compared with the total for all other confounders (17%). Thus, these results suggest a weak influence for maternal LC-PUFAs, both n-6 and n-3, in cognitive development in the offspring. This series of studies has added to the understanding of the mechanisms underlying fatty acid metabolism.

Externalizing behavior was assessed using the Development and Well-Being Assessment at age 7 years (n = 8242); children with a history suggestive of attention deficit hyperactivity disorder (ADHD) and/or conduct disorder were identified.^33^ Although numbers were very small in each diagnostic category, these outcomes were examined in relation to intake of n-3 LC-PUFAs from fish during pregnancy and when the child was aged 3 years (low/high intake) as well as of breastfeeding (none/any) using multivariate stepwise models.^33^ There were no associations of any of these dietary measures with likely diagnosis of attention deficit hyperactivity disorder either before or after adjustment. There was evidence of an unadjusted association between conduct disorder and n-3 LC-PUFA intake during pregnancy and with breastfeeding, but this was completely removed once SEB factors were taken into account.

**Figure 1:**
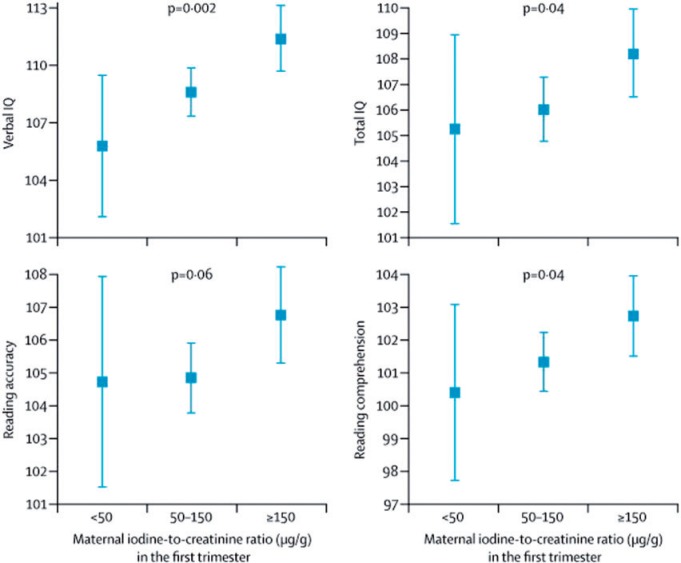
**Means (95% CIs) for child cognitive outcomes according to maternal iodine status in the first trimester.** Values are adjusted for the effect of confounders, including child’s sex, birth weight, preterm delivery, parity, maternal and paternal education, breastfeeding, and n-3 LC-PUFA intake during pregnancy. Child verbal and total IQ were assessed at age 8 years, and reading accuracy and comprehension were assessed at age 9 years. *Abbreviations:* CI, confidence interval; IQ, intelligence quotient; LC-PUFA, long-chain polyunsaturated fatty acid. Reproduced from Bath et al. (2013)^34^ with permission.

The possibility that other nutrients supplied by fish in the diet could be active in neuro-cognitive development was investigated in a study focusing on iodine. Currently in the United Kingdom, but not the rest of Europe, there is evidence of mild-to-moderate iodine deficiency in the population.^34^ Iodine is a major component of thyroid hormones, which are important for fetal brain and neurological development. Urinary iodine concentrations, adjusted for creatinine to account for urine volume, were measured in spot urine samples collected in early pregnancy from 1040 ALSPAC mothers.^34^ Children of mothers with iodine concentrations <150 µg/g, (classified as mild-to-moderately iodine deficient) were compared with those of mothers with concentrations ≥150 µg/g for IQ at age 8 years and reading skills at age 9 years in fully adjusted analysis (Figure 1). Low and mild-moderately low maternal iodine status were both independently associated with an increased risk of suboptimal scores for verbal IQ and reading ability (Figure 1).^34^ These analyses had been adjusted for n-3 LC-PUFA intake from fish consumption during pregnancy, suggesting that the iodine content of fish may partly account for its association with neurocognitive development.

The association between maternal intake of vitamin B_12_ during pregnancy and child IQ was also investigated, and a small association was found with minimal adjustment.^35^ This relationship was greatly attenuated when adjusted for confounders such as maternal education and abolished on further adjustment for birth weight and breastfeeding duration. It is possible that this was an over-control because both birth weight reduction and reluctance to breastfeed may be on the pathway between vitamin B_12_ deficiency and IQ. However, because genetic effects of alleles that increase B_12_ status were only weakly associated with IQ, the authors concluded that maternal B_12_ status is unlikely to have an important causal relationship with offspring IQ.

These analyses underline the importance of fully accounting for factors, such as SEB, which may be associated with both dietary intakes and the behavioral or cognitive outcomes. Lack of appropriate adjustment may account for the inconsistent results from studies in this area of investigation.

### Eczema, asthma, and atopy

It has been suggested that maternal diet during pregnancy may be related to the development of atopic disorders such as eczema and asthma in childhood. The possibility that concentrations of n-3 and n-6 LC-PUFAs measured in maternal and umbilical cord red blood cell membranes may be associated with wheezing and eczema assessed by parent-completed questionnaire in early childhood (at ages 18 months and 3 years) was investigated.^36^ There was some very weak evidence of associations of some ratios of fatty acids in cord blood but not maternal blood with eczema (the ratio of arachidonic acid to eicosapentaenoic acid was positively associated [adjusted OR per doubling, 1.14; 95% CI, 1.00–1.31; *P* = 0.04]) and later-onset wheeze (the ratio of linoleic acid to α-linolenic acid was positively associated [adjusted OR per doubling, 1.30; 95% CI, 1.04–1.61; *P* = 0.02]).^36^ So far, there have been no further studies to determine whether similar associations are found with longer-term history of asthma or markers such as bronchial hyperactivity.

Trace element contents of cord samples were also explored in relation to these outcomes.^37^ Amounts of selenium, zinc, copper, manganese, magnesium, iron, lead, and mercury were measured. The analysis confirmed a relationship between maternal fish consumption and mercury concentrations, which were strongly associated with both oily and white fish consumption assessed in the FFQ. There was some evidence to suggest that selenium concentrations in the umbilical cord were negatively associated with persistent wheeze (adjusted OR, 0.67; 95% CI, 0.45–0.99) and iron concentrations were negatively associated with late-onset wheeze (adjusted OR, 0.86; 95% CI, 0.75–0.99) and eczema (adjusted OR, 0.90; 95% CI, 0.83–0.98) in early childhood.^37^ None of the other minerals were related to these outcomes. It is likely that pregnant women are not all achieving the recommended intakes of selenium or iron,^8^ but further research is needed to confirm these findings before recommendations can be made.

Another set of analyses investigated the development of eczema, asthma, and atopy in relation to the dietary patterns found during pregnancy.^38^ Strong univariate associations were found with the health-conscious dietary pattern for both eczema (positive) and wheeze (negative) and the processed dietary pattern for wheeze (positive), but these associations were attenuated to the null on adjustment. Examples of these associations for persistent wheeze were unadjusted OR of 0.78 (95% CI, 0.70–0.87) and adjusted OR of 1.00 (95% CI, 0.86–1.16) with the health-conscious pattern score and unadjusted OR of 1.27 (95% CI, 1.15–1.40) and adjusted OR of 1.00 (95% CI, 0.88–1.13) with the processed pattern score.^38^ This attenuation is likely to be due to the strong association of the patterns with socioeconomic variables, which are independent determinants of eczema (affluence – positive) and wheeze (low maternal age, smoking during pregnancy, living in rented housing – positive). Associations with atopy (response to skin prick tests) at age 7 years were also not robust to adjustment.^38^

An additional exploration researched the possibility that folate intake and supplementation during pregnancy and/or the methyltetrahydrofolate reductase (*MTHFR*) *C677T* genotype of the mother or child may be related to childhood allergy or atopy, as had been found in a Danish study in adults.^39^ In the children at age 7 years (n = 5364), the prevalence of atopy (positive skin prick test) was 20% and asthma (doctor diagnosed or wheeze present) 10%; there was no association between these outcomes and either the child’s *MTHFR* genotype or maternal dietary folate intake during pregnancy. In the mothers (n = 7356), self-reported allergy was 42% and asthma 11%; again, there was no evidence of an association with the mother’s own *MTHFR* genotype.^39^ Taken together, there was no suggestion that impaired folate metabolism is associated with allergy in this population.

Advice on healthy eating during pregnancy may increase nutrient intakes but the evidence to date suggests that this is likely to have only very limited (if any) effect on preventing the development of eczema, asthma, or atopy in the offspring.

### Obesity development in offspring

The association between maternal prepregnancy BMI and offspring BMI could be due to an intrauterine programming effect or to genetic or environmental effects or a combination of all three. To assess these relationships, maternal and paternal offspring pairs were formed and BMI associations compared when the child was aged 7 years.^40^ A stronger association between maternal–offspring than paternal–offspring pairs would imply an intrauterine effect, which might be expected to accumulate over generations. In ALSPAC, there was no difference in strength between the maternal and paternal associations with offspring obesity (n = 4654 complete trios). These results suggest that the associations between parental and offspring obesity are likely to be due to shared genetic and environmental characteristics rather than intrauterine effects, with shared diet being a plausible contributor.^40^ These possibilities are explored further in the review of childhood diet in ALSPAC, also published in this supplement.^78^

Maternal prepregnancy weight and weight gain during pregnancy were investigated in relation to offspring body composition and cardiovascular risk factors at age 9 years (n = 5154)^41^; however, pregnancy diet was not included in this analysis. Greater prepregnancy weight was associated with greater offspring adiposity (higher BMI, waist circumference, and fat mass) and more adverse cardiovascular risk factors (higher systolic blood pressure and lower high-density lipoprotein cholesterol concentrations). Greater weight gain in early pregnancy up to 14 weeks was associated with increased adiposity, particularly if the mother gained more than 500 g/week. For weight gain between 14 and 36 weeks, only the higher weight gain (>500 g/week) was associated with offspring adiposity.^41^ Adverse cardiovascular risk factors in the offspring were associated with greater pregnancy weight gain, and this was mediated through child adiposity. The possible contribution of gestational diabetes/glycosuria to offspring over weight and obesity at age 9–11 years was investigated in 6842 mother–offspring pairs.^16^ The unadjusted associations found with gestational diabetes were attenuated by adjustment for maternal prepregnancy BMI; however, there were independent associations with glycosuria (≥2 episodes during the pregnancy); the adjusted ORs were 1.35 (95% CI, 1.00–1.82) for general overweight/obesity and 1.31 (95% CI, 1.00–1.72) for central obesity (top 10% of waist circumference) in the children of mothers with glycosuria compared with those of mothers with no sign of diabetes/glycosuria.^16^ Future investigation of dietary associations with these variables could be informative.

Environmental factors that modify DNA methylation at critical time points can affect gene expression and cellular function, and this type of epigenetic change has been speculatively linked to adult obesity. Some nutrients, such as folate, can supply methyl groups to the methylation process; therefore, maternal supplementation with folate and dietary intake of folate during pregnancy may be important and, in theory, could be related to offspring obesity. Childhood body composition at age 9 years was investigated in relation to folate supplementation and dietary intake during pregnancy (n = 5783),^42^ but no evidence that intrauterine exposure to folate influences body composition, including fat mass, at this age was found. A genetic variant in the maternal *MTHFR C677T* genotype was used in the method of Mendelian randomization^79^ to further assess the possible relationship with folate. The *MTHFR* gene influences the availability of methyl donors during pregnancy and, thus, affects DNA methylation and may be expected to be related to offspring obesity in the same way as folate. The use of a genetic marker in this way can overcome the biases often found in observational studies. Again, no relationship was found between the genotype and childhood body composition, thus mirroring the null finding with folate intake.^42^

### Maternal diet in relation to offspring diet in childhood

Associations between maternal prenatal diet and offspring diet in relation to fruit and vegetable intake^43^ and the consumption of a varied diet^44^ have been investigated in collaboration with European partners in the HabEat consortium.^80^ Data on maternal intake during pregnancy in France, obtained via FFQ, were also available (the EDEN study^80^). Maternal and child fruit and vegetable intake and healthy plate variety score (a measure of variety of healthy foods consumed)^44^ were categorized in the same way in the 2 studies. Child’s diet was assessed by FFQ at ages 2 years and 3 years in EDEN and ages 2, 3, and 4 years in ALSPAC. At all ages, a child’s intake of fruit and vegetables was strongly related to maternal intake.^43^ In ALSPAC, the children whose mothers consumed greater amounts of fruit and vegetables were more likely to eat fruit and vegetables at least 3 times a day: adjusted ORs were 3.10 (95% CI, 2.70–3.70), 3.10 (95% CI, 2.70–3.70), and 5.50 (95% CI, 4.70–6.30), at ages 2, 3, and 4 years, respectively. The results were similar in France. The child’s diet variety score was higher if their mother had a higher variety score in both the United Kingdom and France (<0.001 at each age in both cohorts).^44^ Nutrient intakes were not assessed in these analyses; however, the results provide evidence that, in terms of foods consumed, a mother’s diet is positively associated with her child’s diet.

The associations between overall maternal energy and macronutrient intakes during pregnancy and later childhood diet were investigated using dietary data collected from the children at age 10 years (by food record). Maternal and paternal diets, assessed by FFQ when the child was aged 4 years, were incorporated into the analysis to try to distinguish between intrauterine and family dietary relationships.^45^ More than 5000 mother–child pairs and 3000 father–child pairs were available. There was strong evidence of under-reporting of dietary intake in the offspring, so the main results were adjusted for this.^45^ Greater maternal pregnancy macronutrient intakes (protein, fat, and carbohydrate) were associated with greater child intakes of the same nutrients. Associations between maternal–child intakes were stronger than for paternal–child intakes, and maternal pregnancy–child associations were stronger than those with the later maternal diet.^45^ A child’s energy intake at age 10 years was positively associated with the child’s fat mass, as were mutually adjusted protein, fat, and carbohydrate, with fat intake being the strongest predictor. This was only evident once under-reporting was considered. Maternal pregnancy diet was not strongly associated with the offspring’s fat mass.^45^ This pattern of associations suggests there may be intrauterine effects of maternal diet during pregnancy that may program a child’s appetite.^45^

## DISCUSSION

This review has brought together the results of the investigations carried out using ALSPAC data in relation to diet during pregnancy. The dietary data collected in the ALSPAC using the FFQ completed by the women at 32 weeks of pregnancy have been used by a variety of experts in various disciplines, including psychiatrists and psychologists, endocrinologists and nutritionists, epidemiologists, and pediatricians. It was the first of the longitudinal cohort studies in Europe to start during pregnancy and include a measure of diet during pregnancy. More than 20 European population-based birth cohorts with dietary information collected during pregnancy have followed the ALSPAC lead, including the Southampton Women’s Study in the United Kingdom^81^. The focus of the ALSPAC publications to date has been on the mental health of the pregnant woman and aspects of the health and development of her offspring; combining them in one review has emphasized the value of a prebirth cohort study with comprehensive longitudinal data collection. The key findings are listed in Box 1.

Box 1 Key findings in brief

- Eating fish/seafood during pregnancy was associated with beneficial effects on the development of the brain and eyesight of the child
- Women who ate fish/seafood during pregnancy showed fewer symptoms of depression or anxiety than those who ate no fish
- Higher maternal educational attainment was related to better quality of diet consumed during pregnancy
- Maternal smoking during pregnancy and more financial difficulty were related adversely to the quality of the diet consumed during pregnancy
- Some pregnant women had lower-than-recommended dietary intakes of key nutrients such as iron, potassium, magnesium, and folate
- Maternal diet during pregnancy was predictive of offspring diet in childhood
- High maternal prepregnancy weight and greater weight gain during pregnancy were associated with increased fatness and adverse cardiovascular risk factors in offspring in mid childhood

Dietary intakes during pregnancy were mostly adequate, as measured against dietary recommendations with the exception of some key nutrients, in particular iron, magnesium, potassium, and folate.^8^ Relationships of pregnancy nutrient intakes and dietary patterns with several child growth and health outcomes have been explored within ALSPAC; the majority have shown little evidence of important relationships being present with height,^19^ blood pressure,^20^^,^^21^ eczema,^36–38^ asthma,^36–39^ or atopy.^38^^,^^39^ There was evidence of very small positive effects on bone development of the key marginal nutrients magnesium, potassium, and folate;^23^ this finding is supported by the Southampton Women’s Study, in which consumption of a nutrient-dense prudent diet in the third trimester of pregnancy was positively associated with bone development.^82^

Exploration of direct associations of maternal pregnancy diet with birth outcomes found that if the mother ate no fish during pregnancy there was a small persistent association with a higher frequency of intrauterine growth retardation in her offspring compared with mothers who ate fish frequently.^17^ Data from 19 of the European birth cohorts, including the Southampton Women’s Study but not the ALSPAC, were combined in a meta-analysis assessing birth weight and length of gestation in relation to maternal fish intake during pregnancy.^83^ A small but significant increase in birth weight and a slightly lower risk of preterm birth was found with moderate compared with no fish intake; this study did not investigate intrauterine growth retardation, so it did not directly confirm ALSPAC findings. ALSPAC mothers (of boys) who were vegetarian during pregnancy had a greater likelihood of giving birth to a son with hypospadias than omnivorous mothers.^18^ These results imply that foods eaten during pregnancy can affect the physical development of the fetus.

Several publications have confirmed the importance of fish and seafood consumption during pregnancy, with benefits for both the mother and the child emerging. Anxiety and depression during pregnancy may have adverse consequences, with possible effects on delivery and birth outcomes and the later development and behavior of the child. As such, it is of public interest that 2 ALSPAC papers reported that women who ate little or no fish and seafood during pregnancy had increased risk of developing depressive^12^ and anxiety symptoms.^13^ Furthermore, robust evidence was found that the offspring of mothers who did not eat fish or seafood during pregnancy showed poorer neurocognitive development than the offspring of those who frequently ate fish and seafood. This was particularly in relation to visual development,^26^ communication,^27^ and verbal IQ.^28^ In support of these findings, the Danish National Birth Cohort and a prebirth cohort study from the United States (Project VIVA) reported similar associations with maternal fish intake during pregnancy when they studied attainment of developmental milestones at 18 months in Denmark^84^ and child cognition at 2 time points—ages 6 months^85^ and 3 years^86^—in the United States. A plausible theoretical mechanism for these associations relates to the preferential incorporation of n-3 LC-PUFAs (supplied by fish in the diet) into brain cell membranes during fetal development. ALSPAC data was used to explore the genetic underpinning of n-6 and n-3 LC-PUFA concentrations in maternal and offspring blood, showing that both maternal and child genotypes influence fatty acid status during pregnancy.^29^^,^^30^ However, only very weak associations between maternal LC-PUFA concentrations and offspring IQ^32^ were found, suggesting that the strong relationship between fish consumption during pregnancy and offspring cognitive function may have other contributing factors. Fish is a rich source of many nutrients, including iodine. Iodine is a vital component of the thyroid hormones crucial for brain and neurological development, so it is possible that the iodine status of the mother could contribute to the association of fish intake with cognitive development. In ALSPAC offspring, low maternal urinary iodine concentration was associated with lower scores for verbal IQ and reading ability, and this was independent of n-3 LC-PUFA intake from fish (Figure 1).^34^ Considered together, these studies imply that pregnant women should be advised to include fish as part of their diet; this is an important public health message.

However, there are concerns about fish consumption because certain types of fish are contaminated by mercury, which is toxic to humans at high concentrations. The blood mercury concentrations, reflecting the usual UK diet, were quite low,^10^^,^^27^ and ALSPAC data showed that fish consumption contributes to the maternal burden of mercury^10^ and that amounts of mercury were higher in umbilical cord samples if mothers ate fish during pregnancy,^27^ but no evidence was found that these relatively low concentrations of mercury were detrimental to offspring cognitive development.^27^^,^^28^ Project VIVA also found a direct relationship between mothers’ fish consumption and their blood mercury concentrations. ^86^ Higher mercury concentrations were associated with poorer cognitive test performance in the offspring at age 3 years, but higher fish intake was associated with better test scores. Associations were strengthened when both fish and mercury were included in the analysis. These findings are in line with the ALSPAC findings.

It is possible that the associations found between fish consumption during pregnancy and neurocognitive development arose as a result of residual confounding because both fish consumption and some measures of neurocognitive development are related to maternal educational attainment. However, visual development at age 3.5 years was associated with maternal fish consumption but not with maternal education.^26^ Furthermore, when assessing the relationships between maternal fish consumption and childhood IQ, paternal diet showed no independent association and did not attenuate the association with maternal diet substantially.^28^ Taken together these findings indicate that it is more likely that an intrauterine effect of maternal diet is involved in the association with offspring IQ and it is unlikely that maternal education or other social factors are important confounders of this relationship.

Several analyses have confirmed that maternal educational, financial, and smoking statuses are associated with differences in food and nutrient intakes in the maternal diet.^9^^,^^28^^,^^15^ In particular, mothers with the lowest educational attainment were less likely to eat fish and foods associated with a health-conscious dietary pattern than those with the highest educational attainment.^9^ Mothers who smoked and those with financial difficulties ate more processed foods than those who did not smoke or those with no financial difficulties.^9^^,^^15^ The UK Southampton Women’s Study cohort found similar inequalities in women’s diets that were mainly associated with educational attainment.^87^ These diet inequalities could play a role in later health inequalities in the offspring, both in an intrauterine context and later in childhood, particularly as there is evidence that childhood diets are related to mothers’ pregnancy diets.

There was evidence that mothers’ weight status before and during pregnancy is related to later offspring adiposity and, thus, to cardiovascular risk factors in the offspring, with high maternal weight or weight gain being detrimental.^41^ Further investigation is needed to determine whether maternal diet during pregnancy plays any role in these relationships.

There was some evidence that maternal diet during pregnancy is related to the child’s macronutrient intake at age 10 years^45^; maternal pregnancy–child associations were stronger than those with the later maternal diet or paternal diet, suggesting the possibility of an intrauterine affect. This could be through the programming of appetite. However, the associations found between maternal fruit and vegetable intake^43^ and diet variety^44^ and similar measures in their young children are more likely to be due to the copying of parental habits and the availability of particular foods in the household. These suggestions are supported by findings from the Southampton Women’s Study assessing the influence of maternal diet and other characteristics on childhood diet at age 3 years.^88^

It is important to note that almost all publications related to child outcomes in ALSPAC have concerned prepubertal children. It is imperative that analyses be undertaken to assess whether the results, particularly adverse ones, are similar for adolescent and adult outcomes.

### Strengths and limitations

A relatively large cohort of women and their offspring was followed intensively over time with standardized measurements of many important outcomes. Prospective assessment of current diet and other outcomes was used rather than asking participants to look back over several years; thus, recall bias was avoided. However, diet was assessed only once during pregnancy (in the third trimester) due to financial constraints and a prerogative to keep participant burden to a minimum. Therefore, possible dietary changes at different stages of pregnancy were not captured. A self-completed, unquantified FFQ designed for this population was used to assess diet; this is a very cost-effective and well-accepted method of dietary assessment but can lead to biases and inaccuracies. Notwithstanding this, the overall nutrient intakes and types of foods eaten were very similar to those found in a UK national survey of women (nonpregnant) carried out around the same time.^8^^,^^72^ The women in ALSPAC were all from a particular geographical area of England; at recruitment they were reasonably representative of the population in the area. There was a very high participation rate from the cohort during pregnancy, but this dropped off over time and when clinic follow-ups were undertaken; thus, at age 10 years less than half of the original cohort was involved. Furthermore, there was differential dropout of the least educated members of the cohort. It is unlikely that this would have altered longitudinal findings, although statistical power would be diminished.

## CONCLUSION

In interpreting results from associations between prenatal diet and outcomes, it is important to note the strong associations between maternal SEB and diet, especially in the types of foods eaten and dietary patterns, with more processed foods being consumed when socioeconomic status was low. However, with notable exceptions, once these factors were taken into account, there was little evidence of causal relationships between intakes of particular nutrients during pregnancy and birth or childhood health outcomes. The most robust relationship found was between maternal fish/seafood intake during pregnancy and neurocognitive development in the offspring, with fish consumption being beneficial to childhood outcomes. There were other less robust associations with fish consumption, such as lower frequency of maternal depressive and anxiety symptoms during pregnancy and less intrauterine growth retardation. Fish constitute the major dietary source of n-3 LC-PUFAs, and investigation of the genetic background relating to the metabolism of these fatty acids has provided some evidence that they may be involved. These findings do not yet confirm any particular constituent of fish as the active ingredient, but the results with iodine deficiency are suggestive of the involvement of this and possibly other nutrients. Whatever the mechanism, the associations suggest that a recommendation to eat fish regularly during pregnancy is the best advice.
